# Supplementary material for: Ageing Significantly Alters the Physicochemical Properties and Associated Cytotoxicity Profiles of Ultrafine Particulate Matters towards Macrophages
Source: Antioxidants (Basel). 2022 Apr 10;11(4):754. doi: 10.3390/antiox11040754 (PMC9030427; doi:10.3390/antiox11040754)
Supplement: Supplementary file 1 [file antioxidants-11-00754-s001.zip › antioxidants-1626063-supplementary.pdf]

Supporting Information

Ageing significantly alters the physiochemical properties and associated cytotoxicity profiles of ultrafine particulate matters towards macrophages

Xu Yan,[1,2] Yucai Chen,[1,2] Li Ma,[3] Yongchun Liu,[3] Yu Qi,[1,2,*],Sijin Liu[1,2]

1. State Key Laboratory of Environmental Chemistry and Ecotoxicology, Research Center for Eco-Environmental Sciences, Chinese Academy of Sciences, Beijing, 100085, China
2. University of Chinese Academy of Sciences, Beijing, 100049, China
3. Aerosol and Haze Laboratory, Advanced Innovation Center for Soft Matter Science and Engineering, Beijing University of Chemical Technology, Beijing, 100029, China

* Corresponding authors: YQ (yuq@rcees.ac.cn).

Manuscript prepared for *Antioxidants*.

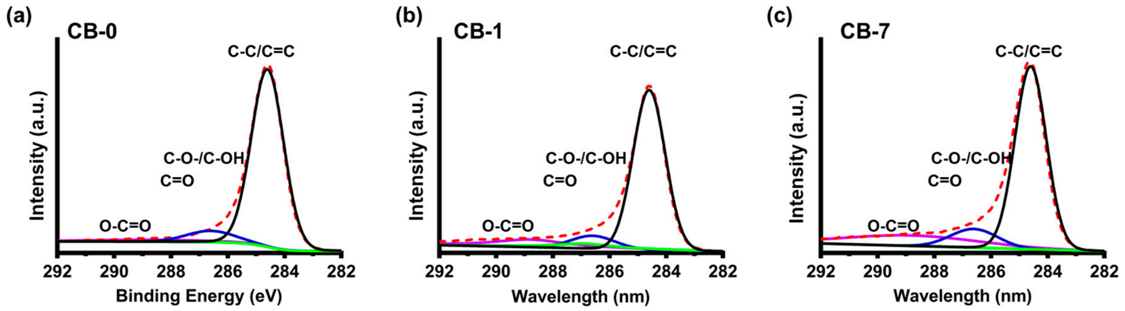

Figure S1 X-ray photoelectron spectroscopy (XPS) spectra with C1 peaks deconvoluted for carbon black (CB) particles. The peaks with the binding energy of 284.6, 286.6, 287.8 eV and 288.8 eV were assigned to the carbon atoms in aromatic rings (C-C/C=C), epoxy/hydroxyl (C-O-C/C-OH), carbonyl (C=O) and carboxyl (O-C=O), respectively. Abbreviations: CB, carbon black.

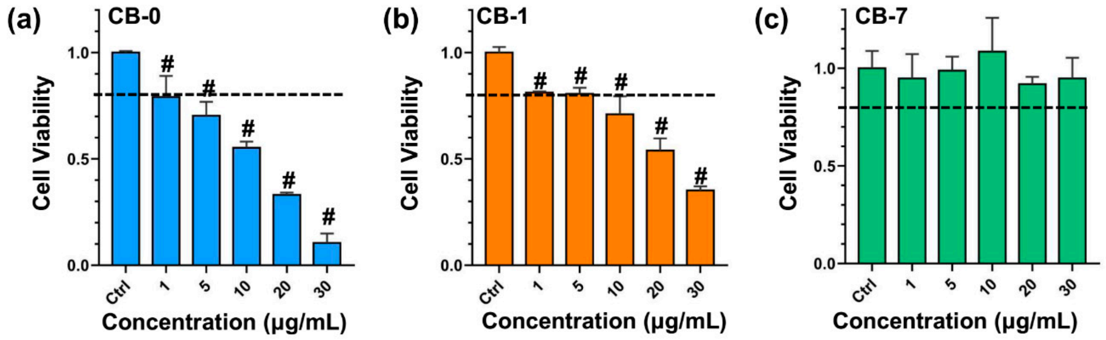

Figure S2 Cytotoxicity of parental and aged CB particles in RAW 264.7 cells. Cytotoxicity of CB-0 (a), CB-1 (b) and CB-7 (c) in RAW 264.7 cells after 24 h treatment at various concentrations (n = 6). All controls are untreated groups. Statistical significance between groups: (#) $P < 0.001$, relative to untreated control. Abbreviations: CB, carbon black.

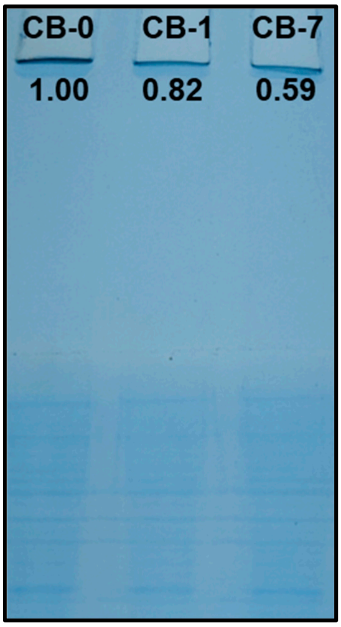

Figure S3 Determination of parental and aged CB particles phagocytosed by macrophages. Coomassie brilliant blue of J774A.1 cells in response to CB particles (10 μg/mL). CB signals intensity was quantified relative to the CB-0 group. Abbreviations: CB, carbon black.

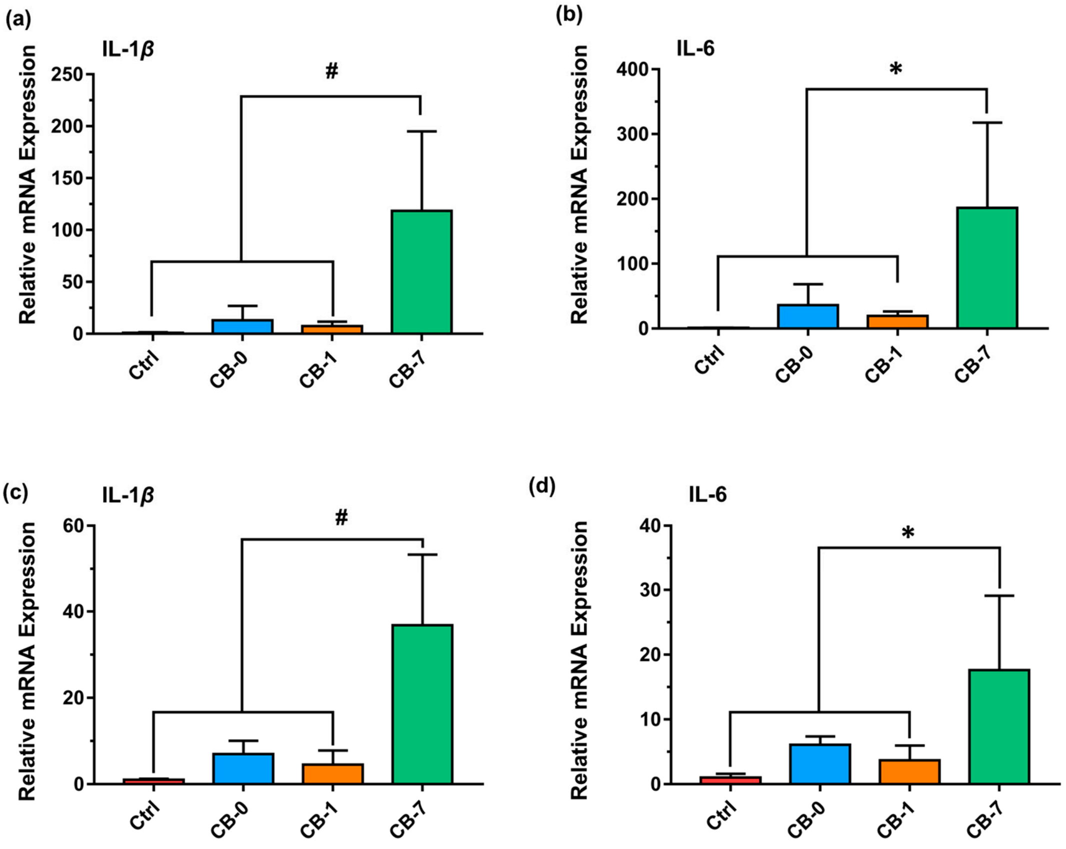

Figure S4 Inflammatory reactions induced by CB particles in macrophages. IL-1*β* and IL-6 expression levels in RAW 264.7 cells (a-b) and BMDMs (c-d) after exposure to CB particles for 24 h, as characterized by qRT-PCR analysis (n = 6). All controls are untreated groups. Statistical significance between groups: (*) $P < 0.05$ and (#) $P < 0.001$, relative to untreated control. Abbreviations: CB, carbon black; IL, interleukin; BMDM, murine bone marrow-derived macrophage.

Table S1 The primers used in real-time quantitative reverse transcription-polymerase chain reaction (qRT-PCR) reactions in the current study.

Genes	Forward (5'-3')	Reverse (5'-3')
IL-1β [a]	GCAACTGTTCCTGAACTCAACT	ATCTTTTGGGGTCCGTCAACT
IL-6 [b]	CTGCAAGAGACTTCCATCCAG	AGTGGTATAGACAGGTCTGTTGG
CyC [c]	AAGAAGGCATGAACATTGTGGAAGC	CGGAAATGGTGATCTTCTTGCTGG

[a] IL-1β: interleukin- 1beta (XM_006498795.5)

[b] IL-6: interleukin-6 (NM_001314054.1)

[c] CyC: cyclophilin (NM_008907.2)
